# Comprehensive analysis of ABA-aldehyde oxidase genes associated with drought and salt stress tolerance in wheat (*Triticum aestivum* L.)

**DOI:** 10.3389/fpls.2026.1897567

**Published:** 2026-08-31

**Authors:** Zilin Fan, Yuyi Ma, Xin Sun, Xu Lu, Yuting Song, Hao Liu

**Affiliations:** Yantai Key Laboratory of Molecular Breeding for High-Yield and Stress-Resistant Crops and Efficient Cultivation/College of Horticulture, Ludong University, Yantai, China

**Keywords:** ABA-aldehyde oxidase (AAO), drought stress, drought-resistant haplotype, protein-protein interaction, salt stress, wheat

## Abstract

**Introduction:**

ABA-aldehyde oxidase (AAO) acts as the final rate-limiting enzyme in ABA biosynthesis, and plays a critical role in plant growth and abiotic stress response. However, comprehensive analysis of *AAO* genes has not been reported in wheat.

**Methods:**

In this study, a total of 30 *TaAAO* genes were identified, and their physicochemical properties, evolutionary relationships, gene structures, *cis*-acting elements, expression patterns, upstream transcription factors, interacting proteins and drought-resistant superior haplotypes were systematically analyzed.

**Results:**

Phylogenetic analysis showed that AAO proteins were divided into five subfamilies, and TaAAOs belonged to subfamilies A, B, C and E. All TaAAOs contained MoCo and FAD domains, and TaAAOs with closer evolutionary relationships exhibited the more similar composition of conserved motifs and exon-intron structures. Promoter analysis showed that ABRE, MeJA-responsive elements, MYC and STRE elements were the most enriched. qRT-PCR assay indicated that most *TaAAO* genes were significantly induced by drought and salt stresses. Especially, *TaAAO2-A, TaAAO3-A, TaAAO4-A, TaAAO5-A*, *TaAAO7-A, TaAAO8-A* and *TaAAO10-A* were significantly induced under both drought and salt stress. The upstream transcription factors and interacting proteins were also analyzed, and Y2H assay showed that TaAAO2-A could interact with Whirly transcription factor TaWHY1-7D. Haplotype analysis showed that *TaAAO2-A-Hap II* was the candidate drought-resistant haplotype with higher survival rate and expression level under drought stress.

**Conclusion:**

These results lay the foundation to study the function of *TaAAO* genes, and provide candidate genes for breeding stress-resistant wheat varieties.

## 1 Introduction

Wheat (*Triticum aestivum* L.) is one of the most widely cultivated staple cereal crops in the world, supplying approximately 20% calories and protein for global human consumption (Sheera et al., 2025). However, escalating climate change has intensified the frequency and severity of abiotic stress, such as drought and soil salinization, resulting in serious yield losses and quality degradation worldwide (Technology, F.S.A., 2022; Chen et al., 2025b; Liao et al., 2026). Therefore, the identification of stress-tolerance genes and exploring their molecular regulatory mechanisms are essential for generating stress-resistant wheat varieties via molecular breeding (Roychowdhury et al., 2024).

The phytohormone abscisic acid (ABA) acts as a key regulator in response to abiotic stresses, including drought and salinity (Hsu et al., 2021; Wang and Xu, 2026). Plants rapidly activate the ABA biosynthesis pathway under abiotic stress to induce a series of biological processes associated with defensive responses, including stomatal closure, accumulation of osmotic regulatory substances, and activation of the antioxidant system, thereby maintaining cellular homeostasis (Marusig and Tombesi, 2020; Muhammad Aslam et al., 2022). ABA-aldehyde oxidase (AAO), belongs to molybdo-flavoenzymes superfamily, is the final rate-limiting enzyme of ABA biosynthesis (Chen et al., 2020). AAO catalyzes the conversion of abscisic aldehyde to active ABA, and also requires FAD (flavin adenine dinucleotide) domain, Moco (molybdenum cofactor) domain and Fe-S (iron–sulfur) clusters as cofactors, thereby regulating core physiological processes, such as seed germination, stomatal opening and closing, and abiotic stress responses (Seo et al., 2000b; Marco et al., 2019; Chen et al., 2020; Wu et al., 2022).

*AAO* genes have been investigated in many plant species, such as *Arabidopsis thaliana* (Seo et al., 2000a, Seo et al., 2004), rice (*Oryza sativa*) (Shi et al., 2021), potato (*Solanum tuberosum*) (Yao et al., 2023), cotton (Liu et al., 2022), peanut (*Arachis hypogaea*) (Long et al., 2019), etc. In *Arabidopsis*, *AtAAO3* plays a major role in ABA biosynthesis in seeds and leaves, while *AtAAO1* and *AtAAO4* slightly contribute to ABA biosynthesis in wild-type seeds, and partially compensate for ABA biosynthesis in *Ataao3* mutants (Seo et al., 2000a, Seo et al., 2004). The loss of *AtAAO2* enhances stress resistance by improving the capacity of *AtAAO3*-mediated aldehyde detoxification (Nurbekova et al., 2024). *AtAAO4* plays a key role in delaying silique senescence by catalyzing aldehyde detoxification (Srivastava et al., 2017). In rice, *OsAO3* gene, the homolog of *AtAAO3*, can be significantly induced by ABA and mannitol treatments. The overexpression of *OsAO3* improves the drought resistance by regulating endogenous ABA level, and the mutation of *Osao3* decreases drought resistance (Shi et al., 2021). After silencing of *GhAAO2* in cotton, the degree of seedling wilting can be alleviated under NaHCO_3_ stress (Liu et al., 2022). The overexpression of peanut gene *AhAAO2* enhances ABA levels and drought tolerance in *Arabidopsis* (Yang et al., 2011; Long et al., 2019).

Although *AAO* genes were identified in several plant species, a systematic genome-wide analysis of the *AAO* gene family in wheat has not been reported. In this study, we identified and comprehensively analyzed *AAO* genes in wheat, including their chromosomal distribution, phylogenetic relationships, gene structures, conserved motifs, promoter *cis*-elements, expression patterns under drought and salt stress, upstream transcriptional regulators, interacting proteins, and haplotype associated with drought tolerance. Our findings provide insights into the evolutionary and functional characteristics of wheat *TaAAO* genes and identify key candidates for improving drought and salt tolerance in wheat molecular breeding.

## 2 Materials and methods

### 2.1 Identification and characterization of *AAO* gene family members in wheat

The reference genome, protein sequences and GFF3 annotation file of wheat were obtained from EnsemblPlants database (https://jun2026-plants.ensembl.org/index.html). The Hidden Markov Model (HMM) of AAO domain (PF01315) was used to retrieve the candidate AAO proteins by searching against the wheat protein sequences using TBtools (Chen et al., 2023a). Then, the candidate AAO proteins were validated using Batch CD-Search (https://www.ncbi.nlm.nih.gov/Structure/bwrpsb/bwrpsb.cgi) and InterPro (https://www.ebi.ac.uk/interpro/) website. The physicochemical properties of TaAAOs were calculated using the Protein Parameter Calc in TBtools software (Chen et al., 2023a). Subcellular localization of TaAAOs was predicted using Plant-mPLoc website (http://www.csbio.sjtu.edu.cn/bioinf/plant-multi/).

### 2.2 Chromosome location and collinearity analysis

Chromosomal localization and paralogous gene pairs of *TaAAO* genes were obtained from the TriticeaeGeneTribe database (http://wheat.cau.edu.cn/TGT), and visualized using the Advanced Circos tool in TBtools (Chen et al., 2023a). The gene duplication events of *TaAAO* genes were analyzed by PLAZA database (https://www.vandepoelelab.be/plaza/). The Ka/Ks ratio of paralogous *TaAAO* gene pairs were calculated to evaluate the evolutionary selection pressure with the Nei-Gojobori (NG) method in TBtools (Chen et al., 2023a).

### 2.3 Phylogenetic analysis

The AAO protein sequences from *A. thaliana*, rice (*O. sativa*), maize (*Z. mays*), cotton (*G. hirsutum)* (Liu et al., 2022) and wheat (*T. aestivum*) were used for multiple sequence alignment with ClustalW and phylogenetic tree construction in MEGA11 under default parameters (Tamura et al., 2021). The phylogenetic tree was constructed with neighbor-joining (NJ) method and 1000 bootstrap replicates. The phylogenetic tree was visualized using Evolview service (https://evolgenius.info//evolview-v2/) (He et al., 2016).

### 2.4 Gene structures, conserved motifs and domains analysis

The exon-intron structures of *TaAAO* genes were obtained from the wheat genome GFF3 annotation file. The conserved motifs and domains of TaAAOs were analyzed using MEME program (https://meme-suite.org/meme/) and Batch CD-Search (https://www.ncbi.nlm.nih.gov/Structure/bwrpsb/bwrpsb.cgi), respectively. The exon-intron structures, conserved motifs and domains of *TaAAO* genes were visualized using the Gene Structure View (Advanced) function in TBtools software (Chen et al., 2023a).

### 2.5 Analysis of *cis-*acting elements of *TaAAO*s promoters

The 2000 bp promoter sequences of *TaAAO*s genes were obtained from EnsemblPlants database (https://jun2026-plants.ensembl.org/index.html), and then submitted to the PlantCare website (http://bioinformatics.psb.ugent.be/webtools/plantcare/html/) (Lescot et al., 2002) to analyze the distribution and quantity of *cis*-acting elements in *TaAAO* promoters.

### 2.6 Transcriptome data analysis

The RNA-seq data from root, stem, leaf, spike, grain tissues in wheat cultivar Chinese Spring were obtained from the WheatOmics 1.0 database (http://wheatomics.sdau.edu.cn/) (International Wheat Genome Sequencing, C 2014). For drought stress, the RNA-seq data (BioProject ID: PRJNA1003680) from leaf tissues in wheat seedlings (Chinese Spring) was obtained from our previous study (Liu et al., 2023). For salt stress, the RNA-seq data (BioProject ID: PRJNA632706) from leaf tissues in wheat seedlings (Chinese Spring) was obtained from PPRD website (https://plantrnadb.com/wheatrna/). The TPM (Transcripts Per Million) values were used to analyze the expression patterns of genes.

### 2.7 Quantitative real-time PCR assay

The 7-day-old seedlings of wheat cultivar “Chinese Spring” with uniform leaf size and root length were selected to determine the expression levels of *TaAAO*s under drought and salt stresses by qRT-PCR. For drought stress, the wheat seedlings were treated with 20% PEG6000 (w/v) for 0 h, 24 h and 36 h. For salt stress, the wheat seedlings were treated with 300 mM NaCl solution for 0 h, 12 h, 24 h and 36 h. The leaf tissues of nine wheat seedlings were collected and mixed for each treatment.

Total RNA extraction from samples was performed using the FastPure Universal Plant Total RNA Isolation Kit (Vazyme), and reverse transcription was conducted with the Hifair^®^V one-step RT-gDNA digestion SuperMix for qPCR kit (Yeasen). qRT-PCR assay was carried out using the SGExcelFast SYBR Mixture (Sangon Biotech) reagent kit. The qRT-PCR primers of *TaAAO*s were showed in **Supplementary Table 1**, and *TaActin* gene was selected as a reference control for normalization. All reactions were performed on three technical replicates, and the relative expression level was calculated using the 2^-ΔΔCt^ method (Livak and Schmittgen, 2001).

### 2.8 The upstream transcription factors prediction of *TaAAO*s

The upstream transcription factors of *TaAAO*s were predicted by wGRN database (https://wheat.cau.edu.cn/wGRN/) (Chen et al., 2023b). The co-expression of *TaAAO*s and their upstream transcription factors was analyzed by TBtools (Chen et al., 2023a) based on the transcriptome data of wheat under drought (BioProject ID: PRJNA1003680) and salt (BioProject ID: PRJNA632706) stresses.

### 2.9 Protein interaction prediction of TaAAOs

The interacting proteins of TaAAOs were predicted through the String database (https://cn.string-db.org/). The correlation of expression under drought and salt stresses between TaAAOs and their interacting proteins were analyzed based on the transcriptome data (BioProject ID: PRJNA1003680 and PRJNA632706), and the correlation heatmap was drawn by OmicShare tools (Mu et al., 2024).

### 2.10 Yeast two-hybrid assay

As described previously, a cDNA library was constructed from drought-treated wheat seedlings (Liu et al., 2020). For Y2H assay, the coding sequence of *TaAAO2-A* was cloned into the pGADT7 (AD) vector, and *TaWHY1-7D* gene was cloned into pGBKT7 (BD). Primers used for vector construction were listed in **Supplementary Table 1**. The recombinant plasmids AD-*TaAAO2-A* and BD-*TaWHY1-7D* were co-transformed into the Y2H Gold yeast cells, and then cultivated on selective media SD-Trp/Leu and SD-Trp/Leu/His/Ade supplemented with x-α-gal.

### 2.11 Haplotype analysis associated with drought tolerance

The haplotypes of the *TaAAO2-A* gene were analyzed using resequencing data of 2789 hexaploid wheat cultivars from the WheatUnion database (https://wheat.cau.edu.cn/WheatUnion/), and the two most prevalent haplotypes were designated *TaAAO2-A*-*Hap I* and *TaAAO2-A*-*Hap II*. Transcriptome data of 200 wheat cultivars (CRA016347) under well-watered and drought conditions were downloaded from the Genome Sequence Archive (https://bigd.big.ac.cn/gsa) (Chen et al., 2025a). The seedling survival rate of 400 wheat varieties under drought stress was obtained from published data (Mei et al., 2022).

## 3 Results

### 3.1 Identification of *AAO* genes in wheat

**Figure 1:**
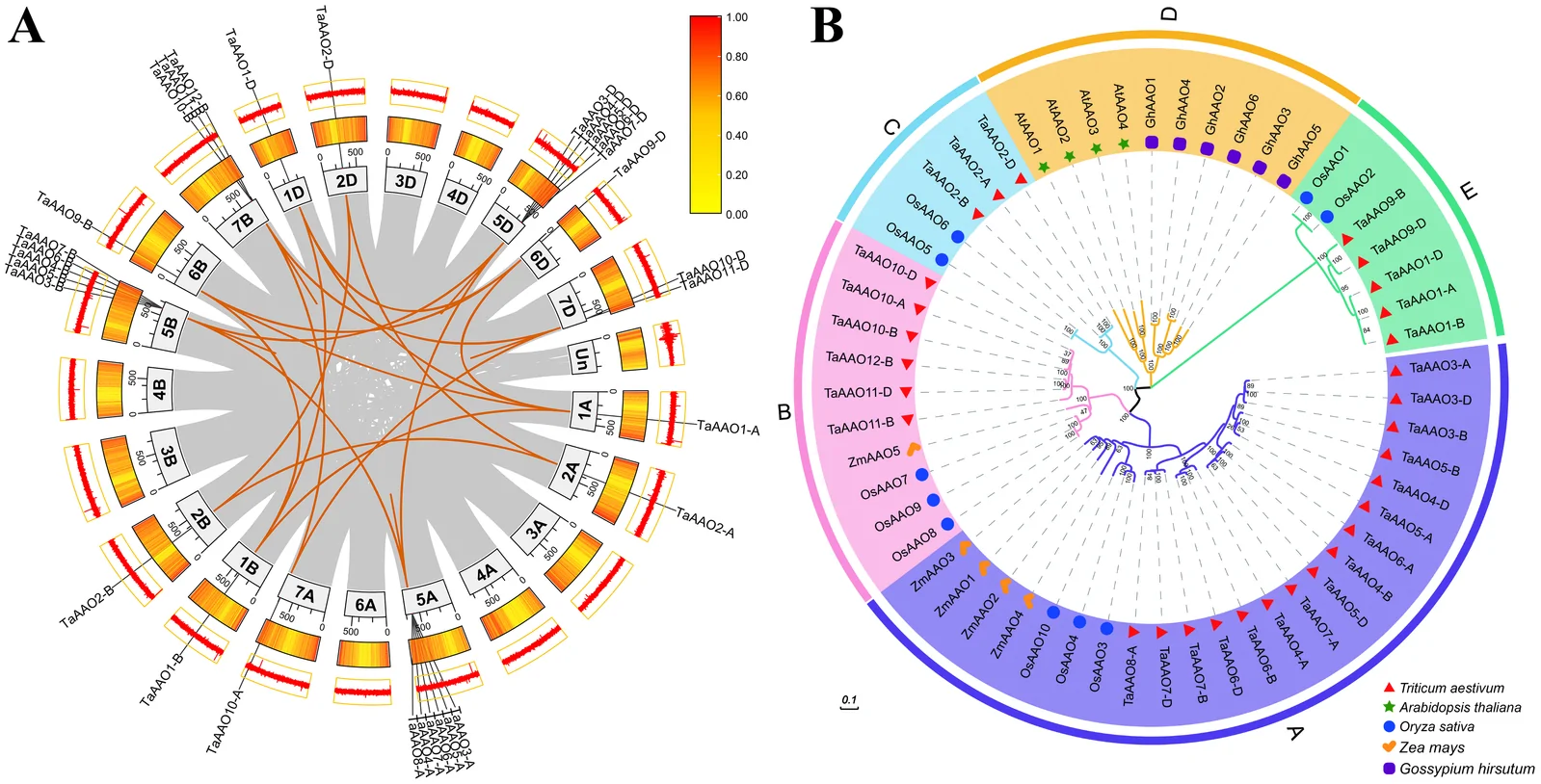
Chromosome localization, collinearity and phylogenetic relationship of *TaAAO* genes in *T. aestivum*. **(A)** Tracks from inside to outside showed chromosomes, GC content, and gene density. The lines in the circle represented the collinearity relationships of wheat genes, and the red lines indicated the collinearity relationships of *TaAAO* genes. **(B)** The neighbor-joining (NJ) tree was constructed using AAO proteins from *A. thaliana*, *O. sativa*, *Z. mays*, *G. hirsutum* and *T. aestivum* with 1000 bootstrap replicates. The AAO protein from different species were marked with different symbols.

A total of 30 *TaAAO* genes were identified in wheat genome and named according to their chromosomal locations and collinearity relationships (**Figure 1**; **Supplementary Table 2**). These *TaAAO* genes encoded proteins ranging from 832 (TaAAO9-B) to 1393 (TaAAO2-A/B) amino acid residues in length, with molecular weights varying from 91.55 (TaAAO9-B) to 150.00 kDa (TaAAO2-B). The isoelectric point (pI) of TaAAOs ranged from 5.55 (TaAAO7-B) to 6.75 (TaAAO12-B), with the Grand Average of Hydropathicity (GRAVY) varying from -0.097 (TaAAO10-D) to -0.004 (TaAAO6-B), suggesting that TaAAOs were acidic and hydrophilic proteins. Subcellular localization predictions suggested that all TaAAO proteins are localized in the chloroplast.

### 3.2 Chromosome location, collinearity and phylogenetic relationship of the *TaAAO* genes

In wheat, a total of 30 *TaAAO*s were unevenly distributed across 14 chromosomes, with each chromosome harboring 1 to 6 members (**Figure 1A**; **Supplementary Table 2**). Chromosome 5A contained the highest number of *TaAAO*s with six members, followed by chromosome 5D and 5B with five *TaAAO*s. Chromosome 7B and 7D had three and two *TaAAO*s, respectively. Chromosomes 1A, 1D, 1B, 2A, 2B, 2D, 6D, 6B, and 7A included one *TaAAO* gene, respectively. The gene duplication of *TaAAO*s was analyzed by PLAZA database, suggesting that *TaAAO1-A/B/D*, *TaAAO2-A/B/D* and *TaAAO9-D* underwent block duplication events, and the other *TaAAO* genes underwent tandem duplicate and block duplicate events (**Supplementary Table 2**). The collinearity analysis revealed that 54 paralogous gene pairs were detected among *TaAAO* genes (**Figure 1A**; **Supplementary Table 3**). The Ka/Ks values of all paralogous gene pairs were less than 1, suggesting that these genes underwent purifying/negative selection to avoid functional divergence (**Supplementary Table 3**).

In order to investigate the phylogenetic relationship of TaAAO proteins, the NJ tree was constructed using AAO proteins from *A. thaliana*, *O. sativa*, *Z. mays*, *G. hirsutum* and *T. aestivum* (**Figure 1B**; **Supplementary Table 4**). The results showed that AAO proteins were divided into five subfamilies, i.e., subfamilies A, B, C, D and E. Subfamilies A, B, C and E contained 16, 6, 3, 5 TaAAO proteins, while no TaAAO proteins were identified within subfamily D. These results suggested that TaAAO proteins might have similar function with the homologous protein in other plant species, and provided insights for the future functional study of TaAAO proteins.

### 3.3 Conserved motif and gene structure analysis of *TaAAO* genes

**Figure 2:**
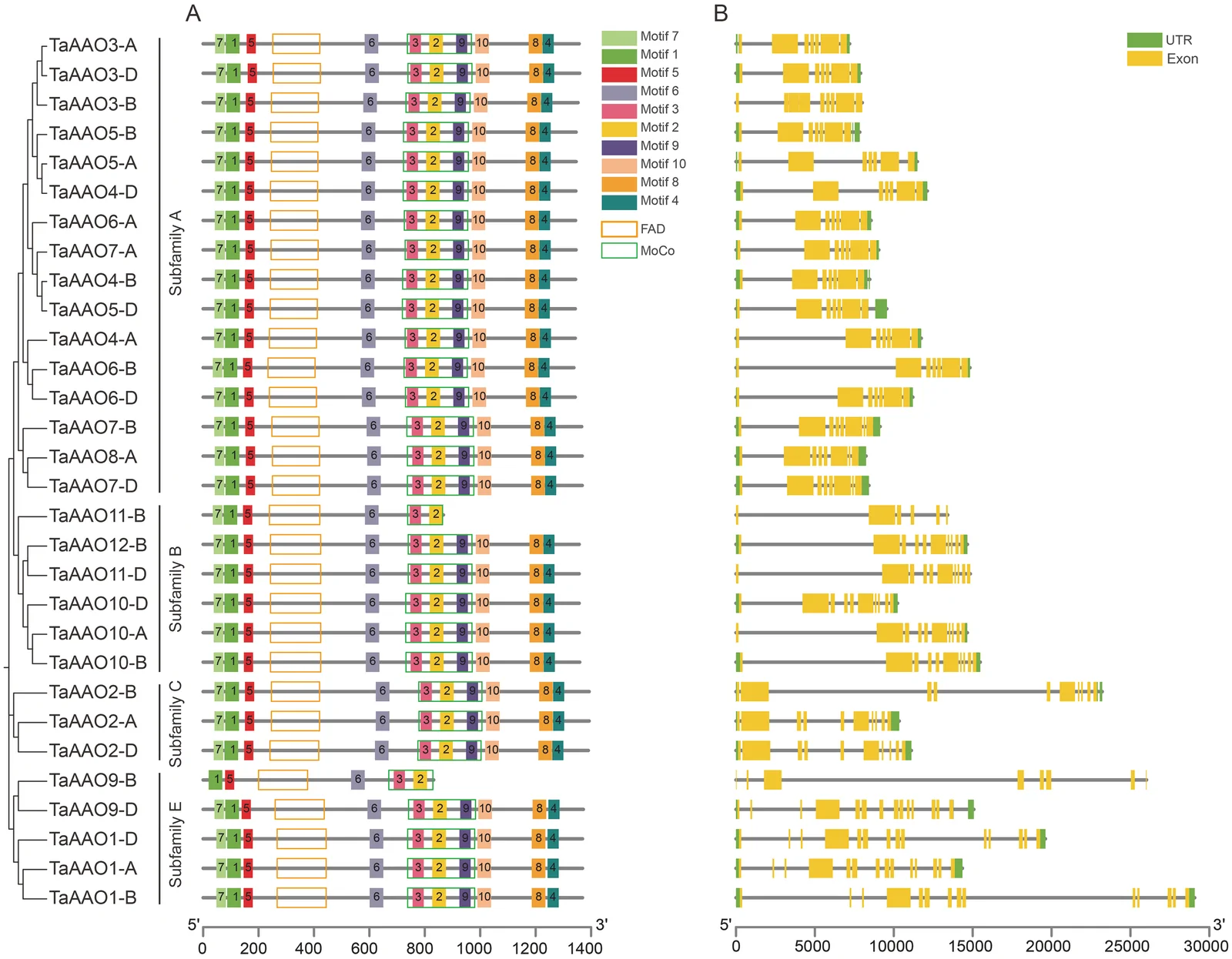
Gene structures and conserved motifs of *TaAAO* gene*s*. The conserved motifs **(A)** and exon-intron structures **(B)** of *TaAAO*s were showed.

To better understand the structural characteristics of the *TaAAO* genes, the conserved motifs and exon-intron structures of 30 *TaAAO* genes were analyzed (**Figure 2**). A total of 10 conserved motifs (Motif 1 to 10) were identified in wheat TaAAOs using the MEME program (**Figure 2A**). Almost all TaAAO proteins contained Motif 1 to Motif 10, whereas TaAAO9-B contained only Motif 1, 2, 3, 5 and 6, and TaAAO11-B was composed of Motif 1, 2, 3, 5, 6 and 7. All TaAAOs contain one MoCo domain and one FAD domain, and the FAD domain of all TaAAOs was composed of Motif 2, Motif 3 and Motif 9 (**Figure 2A**). These results validated the accuracy and reliability of the identified *TaAAO* gene family members.

Exon-intron structures analysis showed that *TaAAO* genes contained 6 to14 introns (**Figure 2B**). Within subfamily A, *TaAAO3-A/D*, *TaAAO4-A/B/D*, *TaAAO5-A/B/D*, *TaAAO6-A/B/D* and *TaAAO7-A* included 7 exons, while *TaAAO3-B* contained 9 exons, *TaAAO7-B/D* and *TaAAO8-A* contained 8 exons. In subfamily B, only *TaAAO11-B* contained 6 exons, *TaAAO10-A/B/D*, *TaAAO11-D* and *TaAAO12-B* possessed 10 exons, respectively. The subfamily C members *TaAAO2-A/B/D* contained 10 exons. The subfamily E members *TaAAO1-A/B/D* and *TaAAO9-D* contained 14 exons, with only *TaAAO9-B* containing 9 exons (**Figure 2B**; **Supplementary Table 2**). These results suggested that TaAAOs with closer evolutionary relationship exhibited the more similar composition of conserved motifs and exon-intron structures.

### 3.4 Prediction of *cis-*acting elements of the promoter

**Figure 3:**
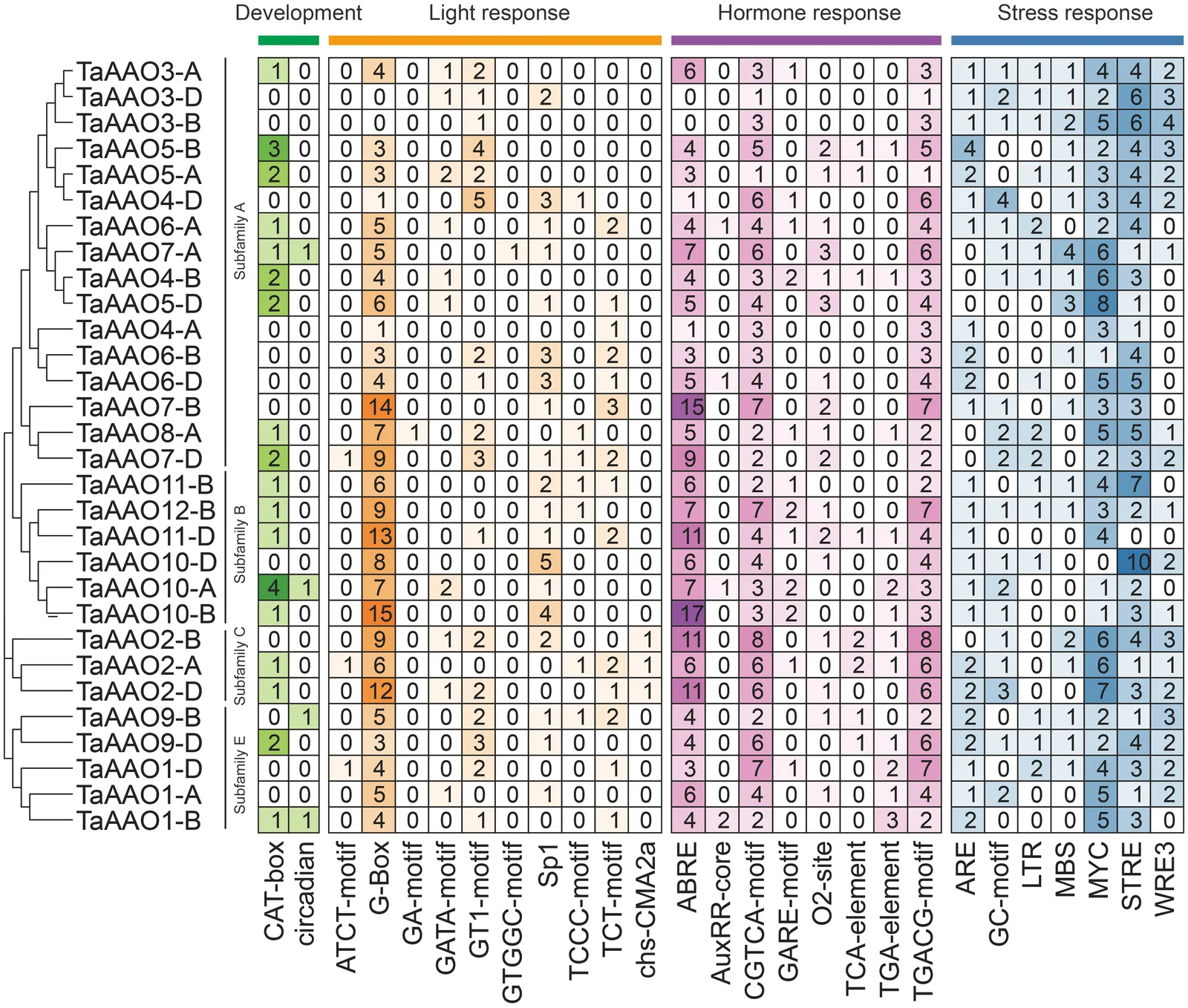
Prediction of *cis*-acting elements in *TaAAO* promoters. The different colors and numbers of grids indicated the counts of *cis*-acting elements in *TaAAO* promoters.

**Figure 4:**
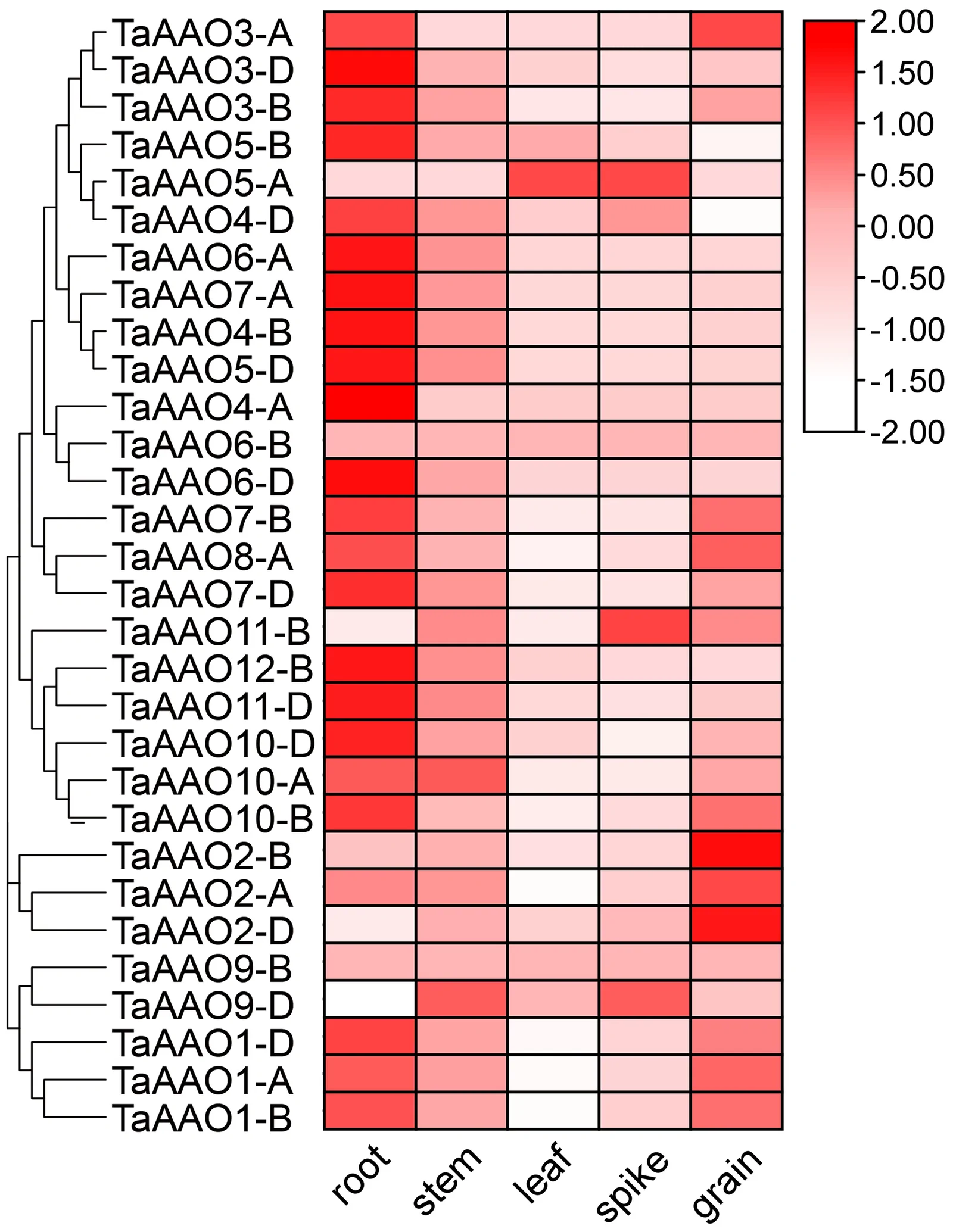
Expression patterns of *TaAAO* genes in different tissues. The TPM values from WheatOmics 1.0 database were used to detect tissue-specific expression levels of *TaAAO* genes in root, stem, leaf, spike and grain.

To detect the potential function of *TaAAO* genes, 2000 bp promoter sequences of each *TaAAO* gene were extracted, and then the *cis*-acting elements were analyzed using PlantCARE database (**Figure 3**; **Supplementary Table 5**). A total of 27 types of *cis*-acting elements related to growth and development, light response, hormone response, and stress response were identified in *TaAAO* promoters (**Figure 4**; **Supplementary Table 5**). Among the growth and development-related elements, CAT-box was the most abundant, which were mainly distributed in the promoters of *TaAAO10-A* and *TaAAO5-B*. Among light-responsive elements, G-box represented the most numerous elements, which were predominantly enriched in the promoters of *TaAAO2-D*, *TaAAO7-B*, *TaAAO10-B* and *TaAAO11-D*. Hormone-responsive elements were also highly abundant with a total count of 489, and ABA-responsive element (ABRE) and MeJA-responsive elements (CGTCA-motif and TGACG-motif) were the most enriched, suggesting the transcription of *TaAAO*s might be modulated by ABA (abscisic acid) and MeJA (methyl jasmonate) signaling pathways. Among the stress-responsive elements, MYC and STRE elements were the predominant *cis*-acting elements in *TaAAO* promoters. Notably, *TaAAO2-A/B/D* and *TaAAO3-A/B/D* contained the highest number of MYC elements. These results suggested that *TaAAO*s might play a central role in response to environmental stresses, especially *TaAAO2-A/B/D* and *TaAAO3-A/B/D*.

### 3.5 Expression patterns of *TaAAO*s in different tissues and drought and salt stress conditions

Tissue-specific expression patterns of the *TaAAO* genes were investigated using publicly available transcriptome data from different wheat tissues, including root, stem, leaf, spike and grain (**Figure 4**; **Supplementary Table 6**). The results suggested distinct expression profiles across different tissues, with root showing the highest overall expression level, followed by grain, stem, spike, and leaf. Distinct expression divergence and obvious tissue-preferential expression patterns were observed among different *TaAAO* members. Most *TaAAO* genes exhibited relatively high transcript abundance in roots. Notably, *TaAAO5-A* was specifically highly expressed in leaf and spike, while the *TaAAO2* (*TaAAO2-A, TaAAO2-B*, and *TaAAO2-D*) showed predominant expression in grain. In addition, homologous copies derived from the A, B and D subgenomes displayed divergent expression patterns. For example, *TaAAO5-A* accumulated high transcripts in leaf, whereas *TaAAO5-B* maintained low expression levels in leaf tissue. Similarly, *TaAAO9-D* was highly expressed in stem and spike, and *TaAAO9-B* exhibited low expression in these tissues. These results suggested that *TaAAO* genes might participate in diverse biological processes during the development of various wheat organs, and functional differentiation has occurred among homologous *TaAAO* copies.

**Figure 5:**
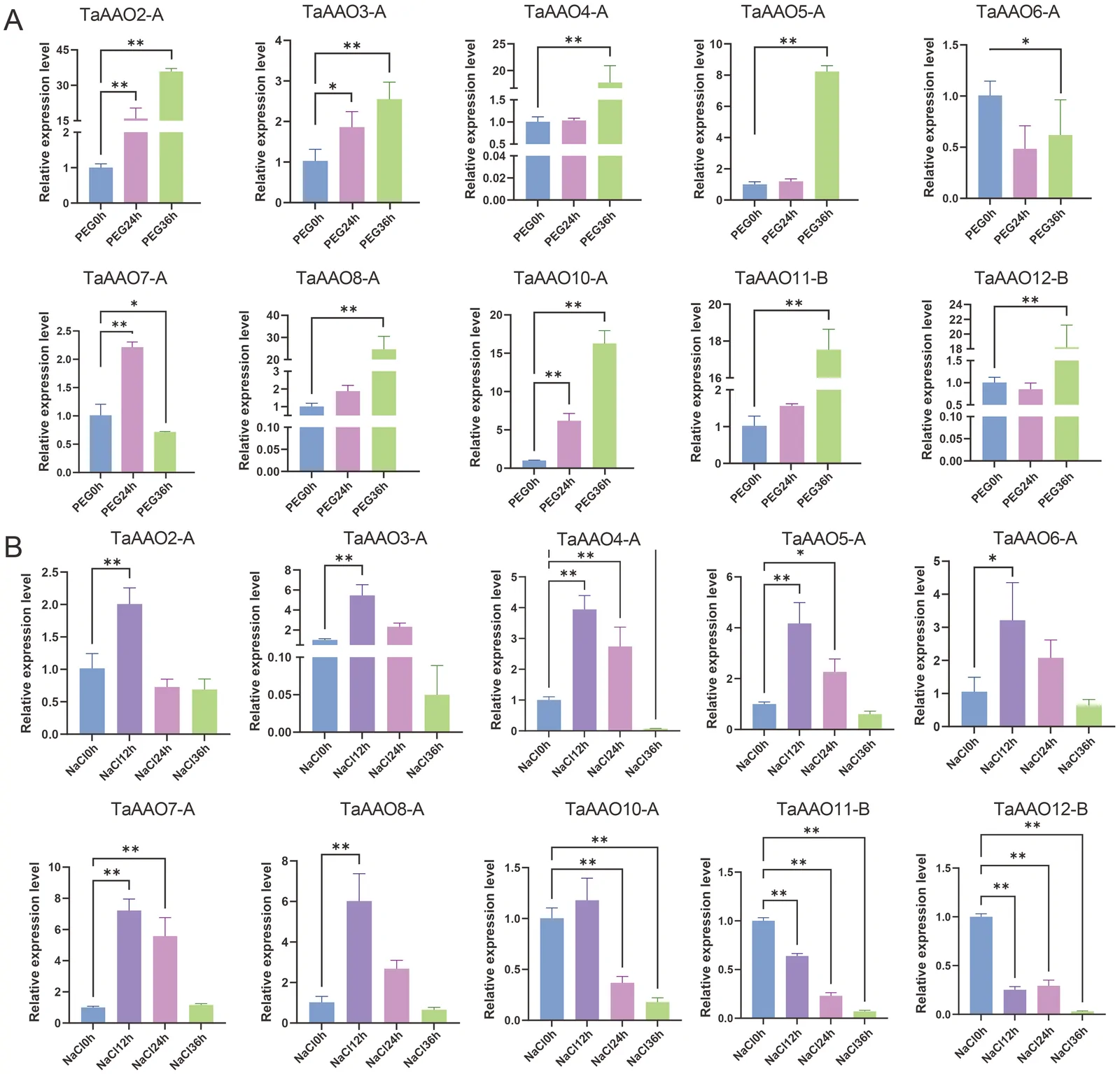
Expression patterns of *TaAAO* genes under drought and salt stresses. qRT-PCR was performed to detect the expression patterns of *TaAAO* genes under PEG-simulated drought **(A)** and salt **(B)** stresses in wheat seedling leaves. The values displayed the mean ± SD. Asterisk indicates significant differences compared with control based on Student’s t-test (*p < 0.05 and **p < 0.01).

To explore the potential roles of *TaAAO*s in response to abiotic stress, the expression profiles of *TaAAO*s were detected under PEG-simulated drought and salt stresses in wheat seedling leaves using qRT-PCR (**Figure 5**). Under drought stress, the expression of most *TaAAO* genes was significantly up-regulated compared to control (**Figure 5A**). Notably, *TaAAO2-A* exhibited the strongest induction, and its expression level peaking at 36 h with approximately 35.85-fold compared to control. Similarly, the expression of *TaAAO3-A*, *TaAAO4-A*, *TaAAO5-A*, *TaAAO8-A*, *TaAAO10-A*, *TaAAO11-B* and *TaAAO12-B* was significantly up-regulated, with approximately 2.25-, 17.73-, 8.23-, 24.68-, 16.28-, 17.53- and 18.22-fold at 36 h compared to control, respectively. *TaAAO7-A* showed increased expression at 24 h with approximately 2.21-fold, and then decreased at 36 h. In contrast, TaAAO6-A displayed down-regulated expression under drought stress.

After salt stress treatment, the most *TaAAO* genes exhibited the similar expression pattern with initial up-regulation and followed by down-regulation (**Figure 5B**). The expression of *TaAAO2-A, TaAAO3-A, TaAAO4-A, TaAAO5-A*, *TaAAO6-A, TaAAO7-A, TaAAO8-A* and *TaAAO10-A* was significantly up-regulated at 12 h under salt stress, with approximately 2.07-, 5.46-, 3.94-, 4.17-, 3.21-, 7.22-, 6.02- and 1.18-fold, respectively. Conversely, *TaAAO11-B*, and *TaAAO12-B* were significantly down-regulated with prolonged salt stress, reaching the lowest expression levels at 36 h, with approximately 0.07- and 0.03-fold compared to control, respectively. These results suggested that *TaAAO2-A, TaAAO3-A, TaAAO4-A, TaAAO5-A*, *TaAAO7-A, TaAAO8-A* and *TaAAO10-A* were significantly induced under both drought and salt stress, suggesting that these genes might contribute to drought and salt stress tolerance in wheat.

### 3.6 The upstream transcription factors prediction of *TaAAO* genes

**Figure 6:**
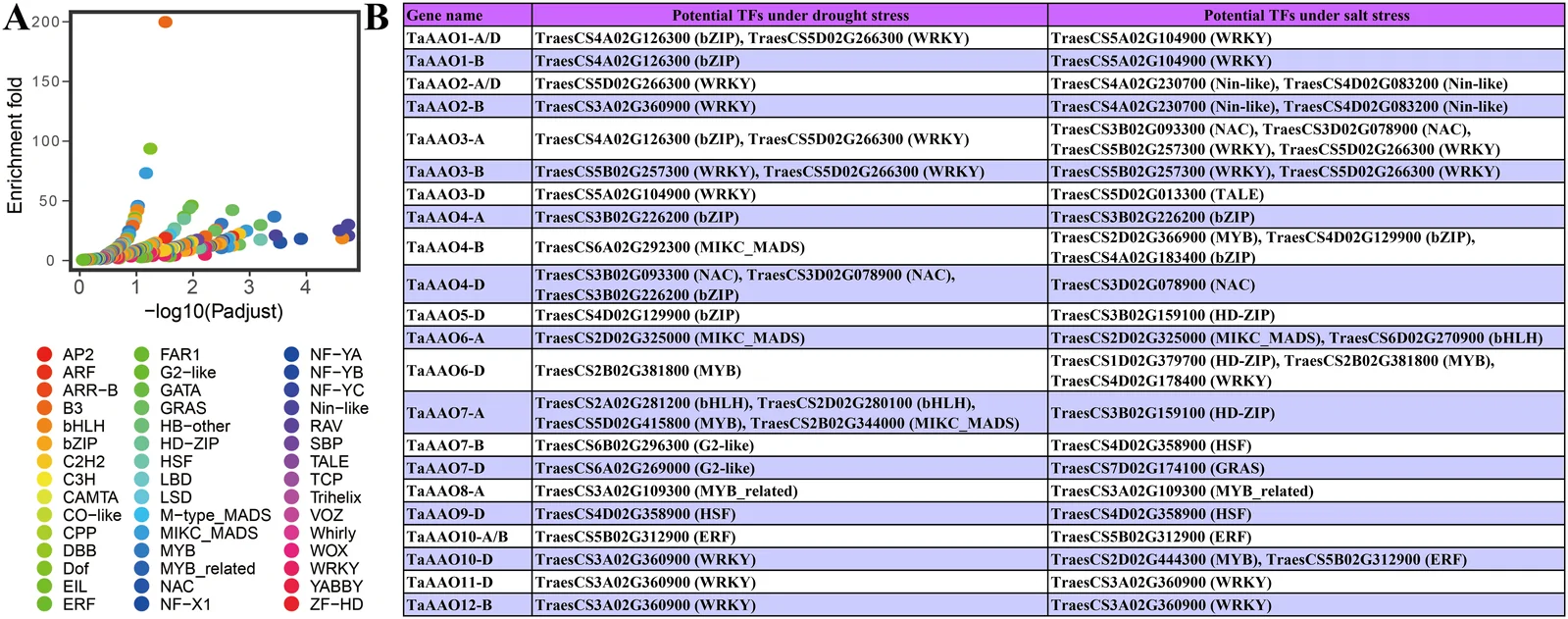
The upstream transcription factors prediction of *TaAAO* genes. **(A)** The upstream transcription factor prediction of *TaAAO*s through wGRN database. **(B)** The TFs that had the similar expression patterns with *TaAAO*s under drought or salt stress were identified.

The upstream transcription factors of the wheat *TaAAO* genes were predicted through wGRN database, and a total of 101 upstream transcription factors belonging to 24 transcription factor (TF) families were determined (**Figure 6A**; **Supplementary Table 6**). MYB, bHLH, and MIKC_MADS transcription factors were the most abundant, accounting for 14.85%, 12.87%, and 7.92% of all identified TFs, respectively (**Figure 6A**; **Supplementary Table 7**). To explore the regulatory mechanism of *TaAAO*s under drought and salt stresses, the co-expression clustering between *TaAAO*s and their upstream transcription factors were analyzed based on transcriptome data (**Supplementary Figures 1**, **2**; **Supplementary Table 8**). The TFs that had the similar expression patterns with *TaAAO*s under drought or salt stress were identified (**Figure 6B**), and these TFs might regulate the transcript levels of *TaAAO*s in response to drought and salt stresses in wheat.

### 3.7 The interacting protein prediction of TaAAOs

**Figure 7:**
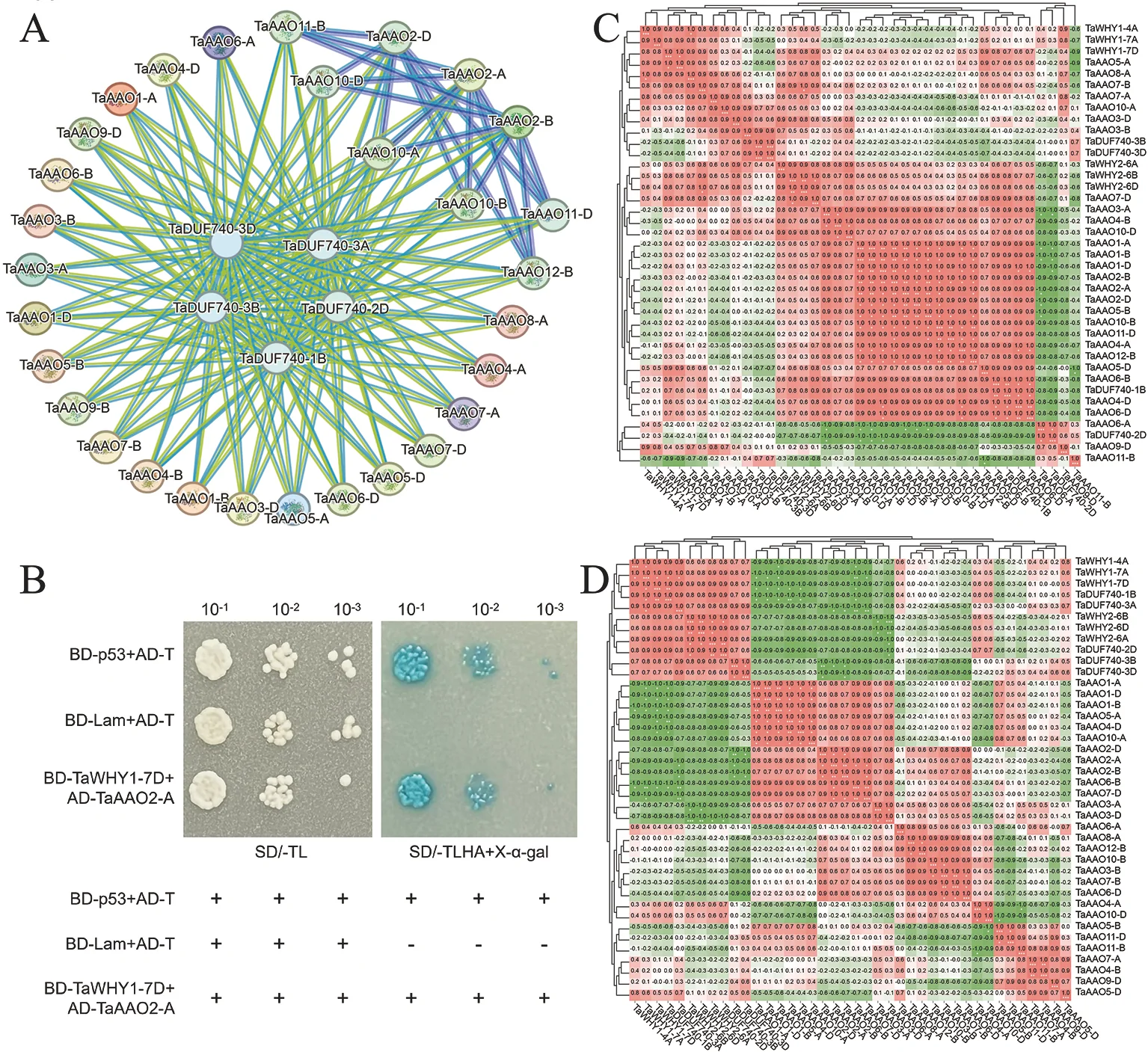
The interacting protein analysis of TaAAOs. **(A)** The interacting protein of TaAAOs were predicted through String database. **(B)** Y2H assay showed TaAAO2-A interacted with TaWHY1-7D. BD-p53+AD-T and BD-Lam+AD-T were as positive and negative controls, respectively. **(C, D)** The correlation analysis between *TaAAO*s and the interacting proteins at the expression level under drought **(C)** and salt **(D)** stresses based on transcriptome data.

The String database and Y2H (yeast two-hybrid) assay were used to identify the interacting protein of TaAAOs (**Figure 7**). The String database analysis results suggested that all TaAAO proteins might interact with OCTOPUS-like protein (TaDUF740-1B, TaDUF740-2D and TaDUF740-3A/B/D). Additionally, TaAAO2-A/B/D might exhibit specific interactions with TaAAO10-A/B/D, TaAAO11-B/D and TaAAO12-B (**Figure 7A**). Y2H assay results suggested that TaAAO2-A could interact with Whirly transcription factor TaWHY1-7D (**Figure 7B**).

Furthermore, the expression correlation between interacting proteins and *TaAAO*s was analyzed based on transcriptome data (**Figures 7C, D**; **Supplementary Table 9**). Under drought stress, *TaAAO2-A/B/D* exhibited positive correlation with *TaAAO10-B/D*, *TaAAO11-D*, *TaAAO12-B*, *TaDUF740-1B* and *TaWHY1-7D* at transcript levels, and displayed negative correlation with *TaAAO10-A*, *TaAAO11-B*, *TaDUF740-2D* and *TaDUF740-3B/D* (**Figure 7C**). Under salt stress, *TaAAO2-A/B/D* exhibited positive correlation with *TaAAO10-A/B* and *TaAAO12-B* at transcript levels, and showed negative correlation with *TaAAO10-D*, *TaAAO11-B/D*, *TaDUF740-1B*, *TaDUF740-2D*, *TaDUF740-3A/B/D* and *TaWHY1-7D* (**Figure 7D**). These results suggested that TaAAOs might be involved in response to drought and salt stresses through interacting with OCTOPUS-like protein and Whirly transcription factor.

### 3.8 Haplotype analysis of *TaAAO2-A* associated with drought tolerance

**Figure 8:**
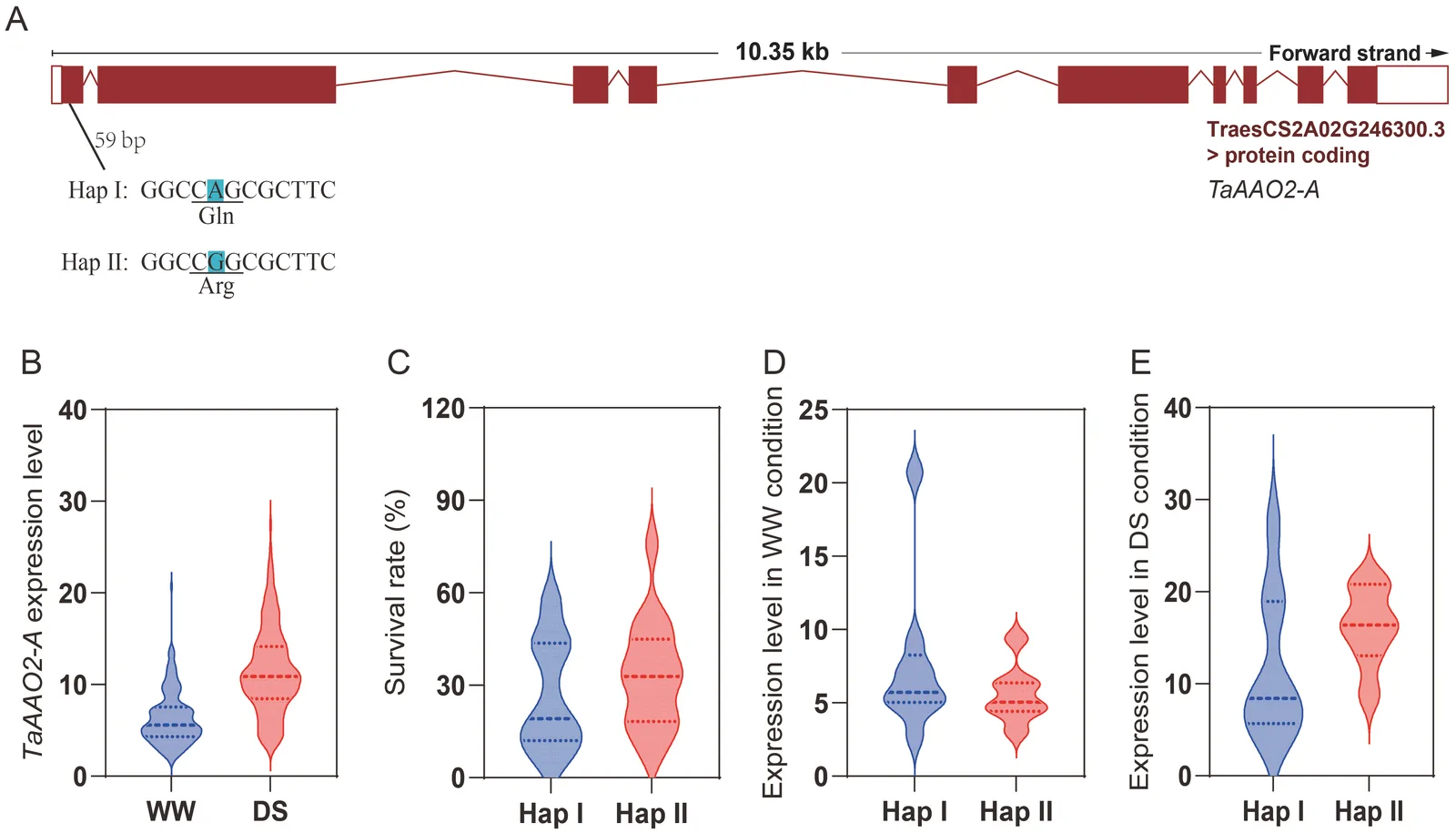
Haplotype analysis of *TaAAO2-A* associated with drought tolerance. **(A)** The main haplotypes of *TaAAO2-A*. **(B)** The expression level of *TaAAO2-A* under well-watered (WW) and drought stress (DS) in 200 wheat varieties. **(C)** The survival rate of 200 wheat varieties under drought stress. **(D, E)** The expression level in WW **(D)** and DS **(E)** conditions of *Hap I* and *Hap II*.

To investigate the relationship between natural variation of *TaAAO2-A* and drought resistance in wheat, haplotype of *TaAAO2-A* was analyzed using Wheat Union database (**Figure 8**; **Supplementary Table 10**). The results showed that a non-synonymous mutation site was identified at 59 bp of *TaAAO2-A* coding region, which caused an amino acid substitution from glutamine (Gln) to arginine (Arg). According to the SNP variants, two main haplotypes of *TaAAO2-A* were identified and named *Hap I* and *Hap II* (**Figure 8A**). *TaAAO2-A* transcript was obviously increased under drought stress (DS) compared with well-watered (WW) condition in 200 wheat cultivars (**Figure 8B**). The seedlings of wheat cultivars with *TaAAO2-A*-*Hap II* had an obviously higher survival rate than *TaAAO2-A*-*Hap I* under drought stress (**Figure 8C**). *TaAAO2-A*-*Hap II* transcript had no obvious change compared with *TaAAO2-A*-*Hap I* under well-watered condition, however, *TaAAO2-A*-*Hap II* transcript was obviously higher than *TaAAO2-A*-*Hap I* under drought stress (**Figures 8D, E**). These results suggested that the *TaAAO2-A*-*Hap II* was the haplotype associated with drought tolerance, providing a valuable genetic resource for breeding drought-tolerant wheat.

## 4 Discussion

Abscisic acid was a phytohormone that regulated plant growth and development, and abiotic stress response, with AAO acting as the rate-limiting enzyme for catalyzing the final step of ABA biosynthesis in plants (Chen et al., 2020). In this study, a total of 30 *TaAAO* genes were identified in hexaploid wheat genome (**Figure 1**), which was more abundant than the *AAO* gene families in diploid *A. thaliana* (Seo et al., 2000a, Seo et al., 2004) and *O. sativa* (Shi et al., 2021). This gene expansion of *TaAAO* was closely associated with the allohexaploid genomic characteristics of wheat (AABBDD) (Levy and Feldman, 2022), indicating that polyploidization had driven the duplication events and functional differentiation of *TaAAO* gene family during wheat evolution.

Previous studies showed that *AAO* genes played important roles in abiotic stress response through ABA signaling pathway (Yang et al., 2011; Long et al., 2019; Shi et al., 2021; Liu et al., 2022). *TaAAO* promoters were enriched with ABRE, MYC and STRE, suggesting that the transcription of *TaAAO*s might be modulated by ABA signaling and abiotic stress (**Figure 3**). In particular, *TaAAO2-A/B/D* and *TaAAO3-A/B/D* contained abundant stress-related *cis*-elements, implying their roles in stress responses. qRT-PCR assay demonstrated that most *TaAAO* genes were significantly induced by drought and salt stresses (**Figure 5**). Notably, *TaAAO2-A*, *TaAAO4-A*, and *TaAAO8-A* were strongly up-regulated under both drought and salt stress, similar to the stress-inducible expression patterns of *AtAAO3* and *OsAO3* (Seo et al., 2000a, Seo et al., 2004; Shi et al., 2021), suggesting that these genes were core candidates involved in wheat drought and salt tolerance. In addition, the expression level of *TaAAO2-A* was obviously increased under drought stress in 200 wheat cultivars, and *TaAAO2-A*-*Hap II* was the candidate drought-resistant haplotype with higher survival rate and expression level under drought stress (**Figure 8**). Although current data indicated that *TaAAO2-A* expression correlated with drought tolerance, direct functional validation using overexpression, CRISPR/Cas9 knockout, or VIGS assays was necessary to confirm the function of *TaAAO2-A* in drought tolerance. For example, overexpression of *OsAO3* improved the drought tolerance in rice, and the knockout mutants *osao3* decreased drought stress tolerance (Shi et al., 2021). In addition, complementary functional validation of *TaAAO* genes was also necessary. For instance, complementation of *TaAAO2-A* in *taaao2-a* mutants can be performed to examine phenotypic recovery.

Y2H assay showed that TaAAO2-A could interact with Whirly transcription factor TaWHY1-7D (**Figure 7**), and TaWHY1-7D could confer drought and salt tolerance in *Arabidopsis* (Liu et al., 2023), suggesting that TaAAO2-A might modulate wheat drought and salt tolerance through interacting with TaWHY1-7D. Although the Y2H assay suggested a potential interaction between TaAAO2-A and TaWHY1-7D, the additional validation assays of protein interaction in planta was still required, such as bimolecular fluorescence complementation (BiFC), co-immunoprecipitation (Co-IP), luciferase complementation imaging (LCI), or pull-down assays. Furthermore, TaAAO2-A and TaWHY1-7D were predicted to be targeted to chloroplasts through bioinformatic analysis (**Supplementary Table 2**), which were the primary organelles for ABA biosynthesis in plants (Milborrow, 2001), suggesting that the interaction of TaAAO2-A with TaWHY1-7D might regulate ABA biosynthesis in response to drought and salt stresses. This speculation required further verification by detecting the protein interaction of TaAAO2-A with TaWHY1-7D in wheat, as well as determination of endogenous ABA levels in wheat under drought and salt stresses. In conclusion, this study provided a theoretical basis for the functional verification of *TaAAO* genes and identified valuable candidate genes and haplotypes for wheat stress-resistant molecular breeding.

## Data Availability

The original contributions presented in the study are included in the article/**Supplementary Material**. Further inquiries can be directed to the corresponding author.

## Supplementary material

Supplementary Figure 1 The co-expression clustering of *TaAAO*s and their transcription factors under drought stress.

Supplementary Figure 2 The co-expression clustering of *TaAAO*s and their transcription factors under salt stress.
